# Severe Sporotrichosis Treated with Amphotericin B: A 20-Year Cohort Study in an Endemic Area of Zoonotic Transmission

**DOI:** 10.3390/jof8050469

**Published:** 2022-04-30

**Authors:** Vivian Fichman, Dayvison Francis Saraiva Freitas, Antonio Carlos Francesconi do Valle, Rogerio Valls de Souza, André Luiz Land Curi, Cláudia Maria Valete-Rosalino, Priscila Marques de Macedo, Andréa Gina Varon, Maria Helena Galdino Figueiredo-Carvalho, Fernando Almeida-Silva, Rosely Maria Zancopé-Oliveira, Raquel de Vasconcelos Carvalhaes Oliveira, Rodrigo Almeida-Paes, Maria Clara Gutierrez-Galhardo

**Affiliations:** 1 Laboratory of Clinical Research in Infectious Dermatology, Evandro Chagas National Institute of Infectious Diseases (INI), Oswaldo Cruz Foundation (FIOCRUZ), Fiocruz. Av. Brasil, 4365, Manguinhos, Rio de Janeiro 21040-900, Brazil; vivianfichman@gmail.com (V.F.); dayvison.freitas@ini.fiocruz.br (D.F.S.F.); antonio.valle@ini.fiocruz.br (A.C.F.d.V.); priscila.marques@ini.fiocruz.br (P.M.d.M.); 2 Medical Service, Evandro Chagas National Institute of Infectious Diseases (INI), Oswaldo Cruz Foundation (FIOCRUZ), Rio de Janeiro 21040-900, Brazil; rogerio.valls@ini.fiocruz.br (R.V.d.S.); andrea.varon@ini.fiocruz.br (A.G.V.); 3 Laboratory of Clinical Research in Infectious Ophthalmology, Evandro Chagas National Institute of Infectious Diseases (INI), Oswaldo Cruz Foundation (FIOCRUZ), Rio de Janeiro 21040-900, Brazil; andre.curi@ini.fiocruz.br; 4 Laboratory of Clinical Research and Surveillance in Leishmaniasis, Evandro Chagas National Institute of Infectious Diseases (INI), Oswaldo Cruz Foundation (FIOCRUZ), Rio de Janeiro 21040-900, Brazil; claudia.valete@ini.fiocruz.br; 5 Laboratory of Mycology, Evandro Chagas National Institute of Infectious Diseases (INI), Oswaldo Cruz Foundation (FIOCRUZ), Rio de Janeiro 21040-900, Brazil; maria.helena@ini.fiocruz.br (M.H.G.F.-C.); fernando.almeida@ini.fiocruz.br (F.A.-S.); rosely.zancope@ini.fiocruz.br (R.M.Z.-O.); rodrigo.paes@ini.fiocruz.br (R.A.-P.); 6 Laboratory of Epidemiology, Evandro Chagas National Institute of Infectious Diseases (INI), Oswaldo Cruz Foundation (FIOCRUZ), Rio de Janeiro 21040-900, Brazil; raquel.vasconcellos@ini.fiocruz.br

**Keywords:** zoonotic, adverse events, *Sporothrix brasiliensis*

## Abstract

Although rare, disseminated sporotrichosis is increasing in several countries. Despite its limiting toxic potential, amphotericin B is the only intravenous antifungal available to treat severe sporotrichosis. We aimed to describe the effectiveness and safety of amphotericin B treatment for severe sporotrichosis. Clinical records of patients with disseminated sporotrichosis at a reference center were reviewed. This study included 73 patients. Most (53.4%) were men and non-white. HIV coinfection was the main comorbidity (52.1%). Most reported contact with cats (76.7%). *Sporothrix brasiliensis* was the causative species. Affected sites were skin (98.6%), osteoarticular system (64.4%), upper airway (42.5%), central nervous system (20.5%), eyes (12.3%), and lungs (8.2%). Median doses of amphotericin B used were 750 mg and 4500 mg for deoxycholate and lipid complex formulations, respectively. Amphotericin B discontinuation occurred in 20.5% due to adverse events, mainly azotemia. The outcomes included cure (52.1%), death due to sporotrichosis (21.9%), death due to other causes (9.6%), and loss to follow-up (8.2%). Survival analysis showed an association between cure and the absence of bone, upper airway, and central nervous system involvement. Amphotericin B is the first-choice treatment for disseminated sporotrichosis; however, the severity of systemic dissemination might predict its response. Favorable clinical results depend on prompt diagnosis, investigation of fungal dissemination, and early therapy initiation.

## 1. Introduction

Sporotrichosis is a ubiquitous mycosis caused by dimorphic fungi belonging to the genus *Sporothrix*. In the last two decades, the state of Rio de Janeiro, Brazil, has become a hyperendemic region of cat-transmitted zoonotic sporotrichosis, and is associated with *S. brasiliensis*, the most virulent species of the genus [1,2].

Cutaneous localized infection is the usual presentation of sporotrichosis; other forms are rare. Unifocal extracutaneous sporotrichosis is locally progressive; primary pulmonary disease follows fungal inhalation, and disseminated disease is associated with hematogenous dissemination. Multifocal disease is most likely to occur in immunosuppressed patients, including patients living with the human immunodeficiency virus (PLHIV) presenting low TCD4+ cell-counts, transplant recipients, patients receiving corticosteroids or tumor necrosis factor antagonists [3,4,5], alcoholism, or diabetes [6].

Although localized cutaneous sporotrichosis is efficiently treated with oral itraconazole, the first-choice agent, management of disseminated forms may be challenging. Amphotericin B (AMB) is the only intravenous antifungal available to treat severe sporotrichosis that is refractory to oral medication [7]. AMB was introduced for clinical use in the 1960s owing to its deoxycholate formulation. Three decades later, lipid formulations were incorporated into the therapeutic arsenal. These formulations are more expensive but better tolerated than deoxycholate. However, they can still be potentially toxic and often require treatment interruption [8].

Disseminated sporotrichosis and refractory cutaneous forms are increasing in the state of Rio de Janeiro and other locations [9,10,11]. To understand the effectiveness and safety of AMB treatment for sporotrichosis, we studied the clinical and epidemiological characteristics of patients with severe sporotrichosis and described their therapeutic experience with AMB.

## 2. Materials and Methods

### 2.1. Study Location, Design, and Patients

We conducted an observational and retrospective study on a cohort of patients diagnosed with sporotrichosis treated with AMB between 1998 and 2018. This study was carried out at the Evandro Chagas National Institute of Infectious Diseases (INI/FIOCRUZ), a reference center for severe and atypical sporotrichosis cases in the hyperendemic area of RJ, Brazil. Atypical cases include hypersensitivity and extracutaneous forms. This study was approved by the Institutional Research Ethical Committee (CAAE 08625819.0.0000.5262). Adult patients diagnosed with sporotrichosis based on fungal isolation and treated with AMB were included in the study.

### 2.2. Definitions

Sporotrichosis was classified as cutaneous localized (lymphocutaneous and fixed forms), cutaneous disseminated, unifocal extracutaneous (single site of involvement or two contiguous sites), and multifocal disseminated (two or more non-contiguous sites) forms. In disseminated forms, organ involvement was defined as the isolation of *Sporothrix* from the respective clinical specimens, such as sputum, bronchoalveolar lavage (BAL), cerebrospinal fluid (CSF), biopsy, or mucosal swab, or utilizing a clinical picture compatible with laboratory and imaging examinations in patients with *Sporothrix* isolation from the skin.

Clinical cure was defined as healing of cutaneous or mucosal lesions, disappearance or stabilization of pulmonary or bone lytic lesions, and sterilization of CSF accompanied by improvement of cellular and biochemical patterns. Patients who were follow-up absentees for at least one month were considered low compliance. The total treatment duration was computed from the initiation of the antifungal agent to the date of cure.

### 2.3. Patient Management

On admission, patients with suspected extracutaneous or disseminated sporotrichosis underwent standard clinical and laboratory evaluations, including HIV serology. In addition, skin samples or samples from other clinically suspicious sites (biopsy, exudate, or aspirate) were collected for routine mycological examination, including fungal culture [12]. In cases of suspected disseminated disease or patients with known immunosuppression, other samples, such as sputum, blood, CSF, and urine, were collected, as well as imaging studies [13]. Anti-*Sporothrix* antibody levels were determined using enzyme-linked immunosorbent assay (ELISA) whenever available at the study center [14]. Molecular identification of *Sporothrix* [15] was performed on strains isolated from the included patients that could be recovered from the mycological collection of the laboratory when the study was conducted.

Patients received AMB deoxycholate 50 mg/day (0.3–1 mg/kg/day), or AMB lipid complex (3–5 mg/kg/day) until clinical improvement. Patients with worsening cutaneous forms despite treatment with itraconazole (100–400 mg/day), terbinafine (250–500 mg/day), or with contraindications for their use were also given AMB. In most severe cases, oral antifungal agents are added to AMB. Since 2013, posaconazole (800 mg/day) has been an oral antifungal option for meningitis with some recalcitrant presentations at our institution [16]. Once the clinical condition stabilized, AMB was prescribed twice a week (usually for 2–3 months) in a day-hospital care center, and then oral antifungal drugs were prescribed to complete a minimum of 12 months of treatment. A new course of AMB treatment was prescribed after worsening or relapse. PLHIV with low TCD4+ cell count received additional antifungal suppressive therapy consisting of itraconazole 200 mg/day until the CD4 count reached ≥200 cells/mm^3^. After treatment, the patients were followed up for 6–12 months.

### 2.4. Adverse Events

The “Division of AIDS (DAIDS) Table for Grading the Severity of Adult and Pediatric Adverse Events” was used in evaluating AMB adverse events (AE) [17]. It provides an AE severity scale ranging from grades 1 to 5, consisting of mild, moderate, severe, and life-threatening AEs and death, respectively.

### 2.5. Data Collection

Clinical, epidemiological, and laboratory data were collected from patients’ medical records. These data were anonymized and de-identified to protect the patients’ privacy and confidentiality.

### 2.6. Statistical Analysis

R Core Team (2021) was used for data analysis. The Shapiro–Wilk normality test was used. In the comparative analysis, according to HIV status and outcome status, the *t*-test or Mann–Whitney test (depending on the distribution pattern) were used for quantitative variables and the chi-square test or Fisher’s exact test was used for the association of qualitative variables. Kaplan–Meier survival curves were used to describe the time to clinical cure, considering each potential risk or protective factors for developing severe forms (sex, clinical presentation, comorbidities, and treatment compliance). Censure was defined as death or loss to follow-up. Finally, a multiple Cox model was used to analyze the time until clinical cure, considering the significant factors in Kaplan–Meier, selected by Wald’s test significance, and controlling by HIV, treatment compliance, and tuberculosis (TB). The crude hazard ratios (HR) and adjusted hazard ratios and their 95% confidence intervals (CI) were used to evaluate the effects obtained by single and multiple covariates Cox models, respectively. Statistical significance was 5%.

## 3. Results

From 1998 to 2018, 5075 patients were diagnosed with sporotrichosis at INI/FIOCRUZ. During this period, AMB was used to treat mycosis in 74 patients. One patient was excluded from this study owing to incomplete medical records, resulting in a data set of 73 patients (1.44% of all sporotrichosis patients). Table 1 summarizes the patients’ clinical and epidemiological data.

Most patients were male (72.6%), non-white (75.0%), and had seven or fewer years of schooling (58.3%). The mean age was 46.06 years. Regarding possible sources of infection, 56 patients (76.7%) reported contact with cats, 41 of which reported that the cat was sick. Scratching or biting was reported by 19 patients. One patient reported contact with a dog with sporotrichosis, and one patient was a gardener. It was not possible to define the possible sources of infection in 15 of the cases. The median time between symptom onset and admission was 90 days (range: 15–2190 days).

Most patients (91.8%) had one or more coinfections or comorbidities. HIV was the main coinfection, followed by alcoholism, diabetes mellitus, tuberculosis (TB), and corticosteroid use. Among the 38 patients with HIV-sporotrichosis coinfection, 10 were diagnosed with HIV during routine investigations for sporotrichosis.

The most common site of *Sporothrix* sp. isolation was the skin (72 patients, 98.6%), followed by mucosa (17 patients, 23.3%), nasal mucosa (*n* = 9), oral mucosa (*n* = 3), both nasal and oral mucosa (*n* = 4), and ocular mucosa (*n* = 1). Other sites of fungal isolation were sputum (11 patients, 15.0%), CSF and blood (6 patients each, 8.2%), synovial fluid (5 patients, 6.8%), lymph node aspirate (4 patients, 5.5%), urine, BAL, and bone marrow (3 patients each, 4.1%) and bone biopsy (2 patients, 2.7%).

From the 73 patients, we recovered 42 fungal isolates, all identified as *S. brasiliensis* by morphological and molecular methods (Figure S1). Anti-*Sporothrix* antibody detection was performed in 34 patients, with 25 (73.5%) positive results.

**Figure 1:**
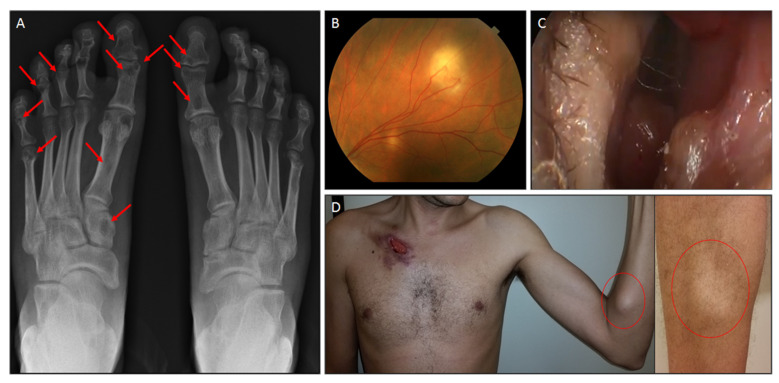
Sporotrichosis multifocal involvement. (**A**) Radiography of the feet of a 36-year-old man, with AIDS and low compliance to treatment. Several rounded to oval osteolytic lesions (red arrows point some) are seen on both feet. (**B**) Fundus image showing yellowish choroidal lesions. (**C**) Right nasal fossa-Infiltration in the right inferior turbinate head, nasal vestibule and superior septal region. (**D**) A 41-year-old previously healthy man with the cutaneous disseminated form of sporotrichosis. Ulcerated lesion surrounded by purplish lesions in the region of the right clavicle. Diffuse painless nodules/masses, here seen in the left forearm and in the right leg (red circles).

Most of the 73 patients (73.9%) presented disseminated sporotrichosis, followed by unifocal disease (skin and bone involvement) and cutaneous disseminated form (Table 1 and Figure 1). Three patients with the cutaneous limited form needed AMB due to low response or contraindication to itraconazole. Two of them were in treatment for TB, which has interactions with itraconazole, and the third was insulin-dependent diabetes with no clinical response after two years of antifungal drugs. The morphological aspects of the skin lesions included ulcers, nodules, and ulcerated nodules. From the upper airways, the lesions consisted of nodules and crusty, infiltrative, and granulomatous lesions. The manifestations of intraocular sporotrichosis were choroiditis (*n* = 3), retinitis (*n* = 2), retinal granuloma (*n* = 1), and uveitis (*n* = 1). Manifestations of external eye involvement included granulomatous conjunctivitis (*n* = 1), episcleritis (*n* = 1) and dacryocystitis (*n* = 1). Osteomyelitis (*n* = 42) and arthritis (*n* = 5) were observed in the osteoarticular system. Central nervous system (CNS) involvement is characterized by meningoencephalitis. Six patients had lung involvement as part of disseminated disease.

The AMB accumulated median doses of deoxycholate and lipid complex formulations are described in Table 1. Forty patients required one course of AMB; nineteen patients, two courses; seven patients, three courses; three patients, four courses; two patients, five courses; one patient, six courses; and another seven courses.

The first course of AMB was followed by itraconazole because of clinical improvement in 71.2% of the patients. In 20.5% of the patients, AMB was suspended due to AE, and 8.2% died during AMB treatment. Patients were either hospitalized (94.5%) or medicated in a day-hospital setting (5.5%) for AMB treatment.

AEs leading to AMB discontinuation in 15 patients included: creatinine elevation (*n* = 12), anemia (*n* = 8), hypokalemia (*n* = 4), phlebitis (*n* = 2), hypomagnesemia (*n* = 1), neutropenia (*n* = 1), thrombocytopenia (*n* = 1), vomiting and chills (*n* = 1), facial edema (*n* = 1), and angioedema (*n* = 1). Considering the severity of AEs reported, they were of grade 4 (60%), grade 3 (16.7%), grade 2 (20.0%), and grade 1 (3.3%). All grade 4 AEs were associated with the deoxycholate formulation. Three patients died after renal AE, two of whom required hemodialysis. Other antifungal drugs used were itraconazole (70 patients, 95.9%), terbinafine (23 patients, 31.5%), and posaconazole (11 patients, 15.1%).

Overall, 38 patients were cured (52.1%), and the lethality due to sporotrichosis was 21.9%, including three patients who had AE with AMB. Six patients died due to other causes, including acute myocardial infarction (*n* = 2), TB (*n* = 2), lymphoma, and bacterial sepsis (*n* = 1 each).

Because PLHIV were the most prevalent group, we compared it with the non-HIV group (Table 2). HIV sporotrichosis patients were younger, predominantly non-white, had a greater proportion of TB coinfection, and accounted for all cases of CNS involvement. PLHIV required a higher AMB dose in both formulations, longer treatment duration (*p* = 0.006), had more AEs due to AMB (*p* = 0.034), and a higher proportion of low treatment compliance (*p* = 0.010). There was no significant difference in anti-*Sporothrix* antibody detection between the PLHIV and non-HIV groups (*p* = 0.050). The cure and other outcomes of interest did not differ between the two groups (*p* = 0.553).

We present the comparison between patients that were cured (52.1%) and those who did not (47.9%) in Table 3. The time between symptom onset and treatment initiation was longer in non-cured patients (*p* = 0.037). They also had a higher frequency of CNS involvement (*p* = 0.041), lower compliance to treatment (*p* = 0.009), and higher doses of AMB (*p* = 0.024). None of the patients with positive blood cultures for *S. brasiliensis* were cured. The exploratory Kaplan–Meier survival analyses showed seven statistically significant variables (sex, HIV, disseminated clinical form, treatment compliance, and bone, upper airway, and CNS involvement). In the multiple Cox model controlled by HIV, treatment compliance, and tuberculosis, a higher ratio of cure was associated with no bone involvement, 2.81 (95% CI: 1.40–5.65); no upper airway involvement, 2.12 (95% CI: 1.05–4.27); and no CNS involvement, 2.70 (95% CI: 0.89–8.14). There were no differences between crude and adjusted HR (Table 4).

## 4. Discussion

Even after more than 20 years of epidemic emergence, sporotrichosis is still not under control in Rio de Janeiro, assuming a hyperendemic status. The increase in cases in this period was accompanied by an increase in severe forms in the reference center, INI/FIOCRUZ. Since 2011, the disease has become notifiable in the state, and other health institutions have been responsible for localized, simple cases. INI remains the primary reference for the most severe cases. All the fungal isolates recovered were identified as *S. brasiliensis*, emphasizing its association with zoonotic transmission and its ability to cause severe cases [18,19].

Most patients were male, non-white, poorly educated, and exposed to other endemic infections such as HIV and TB, sometimes occurring together, as described for vulnerable populations in Brazil [20]. Sporotrichosis is considered a Neglected Tropical Disease by the World Health Organization, as it receives little investment in research, treatment, and prevention, and it mostly affects impoverished communities with low access to the health system [21]. Notably, 13.8% of the patients were newly diagnosed with HIV infection late presenters, evidencing missed opportunities for HIV diagnosis [22]. In addition, standard treatment for TB (rifampicin and isoniazid) interferes with the effectiveness of the oral antifungals. This hampers therapeutic approaches for sporotrichosis.

Diabetes mellitus and alcoholism have known immunosuppressive mechanisms and have been reported as risk factors for severe sporotrichosis [23,24], as herein observed.

A positive blood culture strongly suggests a multifocal form of sporotrichosis [25,26], as well as a positive urine culture, which is related to the hematogenous dissemination of the fungus. Serology for sporotrichosis showed a good yield (73%) in our study cohort, including the PLHIV group. It is a simple and rapid test, offering diagnostic support for extracutaneous and disseminated forms of sporotrichosis, especially when it is difficult to obtain clinical specimens for culture [13,14].

The clinical manifestations of disseminated sporotrichosis presented here, except for skin lesions, were mostly oligosymptomatic, diagnosed through routine examinations of the main sites of fungal dissemination. As expected, the osteoarticular system was the main extracutaneous site of the infection. Additionally, upper airway involvement was a common finding in this population. In a systematic review of HIV-associated sporotrichosis cases, including 58 patients, 20.6% of them had oral or nasal mucosa involvement, or both [27]. Regarding ocular manifestations, we observed a predominance of intraocular disease, which was related to hematogenous dissemination of *Sporothrix* spp. External eye disease, typical of zoonotic transmission, was present in a smaller proportion of patients. It occurs probably through direct eye contact with exudates and respiratory droplets from sick cats [28]. The CNS is affected in a high percentage of immunosuppressed patients, evidencing the potential neurotropism of *S. brasiliensis* [29]. Severe sporotrichosis cases have also been reported in other Brazilian states [30,31].

In this study, we observed the severity profile in PLHIV who had more disseminated sporotrichosis, as previously described [3,9]. There was a higher incidence of multiple-organ involvement, especially in the CNS, which justifies the more frequent prescription of posaconazole. Since TCD4+ cells play a pivotal role in controlling sporotrichosis [32], their low numbers in these patients could explain the dissemination of infection [13,32]. PLHIV had a higher incidence of low treatment compliance, longer treatment times, and several relapses. Studies on PLHIV point out barriers to treatment compliance, such as misuse of substances, depression, and unemployment [33].

AMB is a good option for treating cryptococcosis and histoplasmosis in PLHIV [34,35]. Nevertheless, the lethality of disseminated sporotrichosis is high. The sporotrichosis cure rate achieved with AMB is much lower than those reported for other antifungal drugs [36,37]. However, all parenteral antifungals (fluconazole, voriconazole, echinocandins) except posaconazole have low efficacy against *Sporothrix* spp. [38,39,40], making AMB still the main choice for disseminated and extracutaneous disease. The cumulative AMB doses were very high, reflected in the long hospital stays. Although PLHIV had more severe diseases, their cure rate did not differ from patients without HIV infection. This reinforces the severity of the disease and the limits of AMB in severe sporotrichosis treatment [41]. One explanation for this limitation is that in vitro studies demonstrated good susceptibility of *S. brasiliensis* to oral antifungals, such as itraconazole, posaconazole, and terbinafine, in contrast to the moderate efficacy of AMB [38,40].

We found a high incidence of severe AE (grades 3 and 4) (76.7%) requiring the suspension of AMB (21.4%), especially with the deoxycholate formulation. AMB toxicity prevents its proper prescription, resulting in fungal dissemination and therapy failure [8,42]. Azotemia was the main AE and was associated with death in three patients in our study. Renal dysfunction is a known risk factor for mortality in critically ill patients [43]. Parenteral posaconazole, which has better bioavailability than oral formulations, may be an option for severe forms of sporotrichosis. However, this formulation is not currently available in Brazil. Moreover, itraconazole, which is used after AMB to complete the treatment of disseminated sporotrichosis, has erratic absorption and interacts with several medications, leading to subtherapeutic levels. Itraconazole plasma monitoring is unavailable at the local institutions that manage sporotrichosis, thereby compromising therapeutics.

Delayed diagnosis and consequent late initiation of treatment were more prevalent in the group of patients who did not achieve a cure. Along with delayed diagnosis, multifocal disease and positive blood culture were also present in a higher percentage of patients who were not cured. Late diagnosis may result from difficulties accessing the health system and misdiagnosis of severe sporotrichosis, probably due to its pleomorphic presentations. This was not observed in typical cutaneous forms of sporotrichosis referred to our institute. In these cases, the interval between the onset of cutaneous disease and the time of diagnosis is significantly reduced, reflecting better information about the disease over time [44].

As expected, a significantly higher percentage of patients who were not cured were patients with low compliance and who took higher doses of AMB, without success. We did not study the possible factors associated with low treatment compliance. However, the need to go to the hospital for AMB infusion periodically after clinical stabilization may contribute. Using a retrospective model and only including adult patients from a single center is a limitation of this study.

## 5. Conclusions

The increasing severity of sporotrichosis in endemic areas represents a serious public health and economic problem. Severe sporotrichosis may alert to immunosuppressive conditions and may pose a therapeutic challenge with substantial morbidity and mortality. AMB remains the first-choice treatment for disseminated sporotrichosis, and the severity of systemic dissemination predicts its response. Favorable clinical results depend on prompt diagnosis, the investigation of fungal dissemination, and the early initiation of antifungal therapy. Once treatment begins, attention must be paid to compliance. Future investigations are needed to introduce alternative drugs to treat severe sporotrichosis.

## Figures and Tables

**Table 1:** Clinical and epidemiological data of 73 patients with sporotrichosis treated with amphotericin B at the INI/FIOCRUZ, Rio de Janeiro, Brazil, between 1998 and 2018.

Variable	Status	*N* = 73
Sex	Male	53 (72.6%)
	Female	20 (27.4%)
Age		46.06 [16.18, 83.95] ^d^
Skin color ^a^	Non-white	54 (75.0%)
	White	18 (25.0%)
Schooling ^b^	1–7 years	35 (58.3%)
	>7 years	21 (35.0%)
	Non-literate	4 (6.7%)
HIV		38 (52.1%)
Alcoholism		21 (28.8%)
Diabetes		15 (20.5%)
Tuberculosis		13 (17.8%)
Corticosteroid use		6 (8.2%)
Clinical presentation	Disseminated	54 (73.9%)
	Unifocal extracutaneous	8 (11.0%)
	Cutaneous disseminated	8 (11.0%)
	Cutaneous localized	3 (4.1%)
Affected organ ^c^	Skin	72 (98.6%)
	Osteoarticular	47 (64.4%)
	Upper airways	31 (42.5%)
	Central nervous system	15 (20.5%)
	Ocular	9 (12.3%)
	Lungs	6 (8.2%)
Deoxycholate total dose (mg)		750.00 [50.00, 11,250.00] ^e^
Lipid complex total dose (mg)		4500.00 [200.00, 14,700.00] ^e^
Total treatment duration (days)		696.00 [1.00, 4017.00] ^e^
Outcome	Cure	38 (52.1%)
	Death due to sporotrichosis	16 (21.9%)
	Death due to other causes	7 (9.6%)
	Loss to follow-up	6 (8.2%)
	Still under treatment	6 (8.2%)

^a^ Missing data (*N* = 1); ^b^ Missing data (*N* = 13); ^c^ Several patients had more than one affected organ; ^d^ Mean and range; ^e^ Median and range.

**Table 2:** Comparative characteristics of 73 patients with sporotrichosis treated with amphotericin B, divided into two groups, HIV coinfected and non-HIV coinfected (FIOCRUZ, Rio de Janeiro, Brazil, 1998–2018).

Group		HIV Coinfected Patients	Non-HIV Coinfected Patients	*p* Value
*n*		38	35	
Age ^a^		38.71 [16.18, 55.26]	55.62 [19.45, 83.95]	**<0.001**
Ethnicity	White	5 (13.2%)	13 (38.2%)	**0.028**
	Non-white	33 (86.8%)	21 (61.8%)	
Tuberculosis		11 (28.9%)	2 (5.7%)	**0.013**
Affected organs and systems	Skin	38 (100.0%)	34 (97.1%)	0.479
	Osteoarticular	25 (65.8%)	22 (62.9%)	0.812
	Upper airways	18 (47.4%)	13 (37.1%)	0.478
	CNS	13 (34.2%)	0 (0.0%)	- ^c^
	Ocular	5 (13.2%)	3 (8.6%)	0.712
	Lungs	5 (13.2%)	1 (2.9%)	0.201
Amphotericin B indication	Disseminated infection	34 (89.5%)	23 (65.7%)	**0.022**
	Low response to itraconazole	4 (10.5%)	12 (34.3%)	
Number of AMB cycles ^b^		1.00 [1.00, 7.00]	1.00 [1.00, 2.00]	**<0.001**
Amphotericin B total dose ^b^	Deoxycholate	1465.00 [50.00, 11,250.00]	400.00 [50.00, 8885.00]	**<0.001**
	Lipid complex	6600.00 [1900.00, 14,700.00]	3900.00 [200.00, 11,700.00]	**0.005**
Treatment duration ^b^		834.50 [1.00, 4017.00]	484.00 [7.00, 1899.00]	**0.006**
Adverse effects due to AMB		27 (71.1%)	16 (45.7%)	**0.034**
Other treatments	Itraconazole	37 (97.4%)	33 (94.3%)	0.604
	Terbinafine	12 (31.6%)	11 (31.4%)	1.000
	Posaconazole	10 (26.3%)	1 (2.9%)	**0.007**
Low compliance to treatment		22 (59.5%)	10 (28.6%)	**0.010**
Anti-*Sporothrix* antibody detection	Positive	12 (60.0%)	13 (92.9%)	0.050
	Negative	8 (40.0%)	1 (7.1%)	
Cured		18 (47.4%)	20 (57.1%)	0.553

^a^ Mean and range; ^b^ Median and range; ^c^ *p* value was not calculated due to zero count for non-HIV coinfected patients. *p* values < 0.05 are highlighted in bold.

**Table 3:** Comparative between cured and non-cured patients with sporotrichosis treated with Amphotericin B at the Evandro Chagas National Institute of Infectious Diseases (INI/FIOCRUZ) from 1998 to 2018.

Outcome	Cured	Non-Cured	*p* Value
*N*	38	35	
Symptoms duration until treatment started ^a^ (days)	60.00 [15.00, 2190.00]	120.00 [20.00, 365.00]	**0.037**
Positive hemoculture for sporotrichosis	0 (0.0%)	6 (17.1%)	- ^b^
CNS involvement	4 (10.5%)	11 (31.4%)	**0.041**
Low compliance to treatment	11 (28.9%)	21 (61.8%)	**0.009**
Total dose of amphotericin B (Lipid formulation) ^a^ (mg)	3750.00 [200.00, 10,000.00]	5225.00 [250.00, 14,700.00]	**0.024**
Total dose of amphotericin B (Deoxycholate) ^a^ (mg)	575.00 [50.00, 11,250.00]	1200.00 [50.00, 5390.00]	0.120

^a^ Median and range; ^b^ *p* value was not calculated due to zero count for cured patients; *p* values < 0.05 are highlighted in bold. CNS: Central nervous system.

**Table 4:** Multiple Cox regression model of possible predictors to cure, of the patients with severe sporotrichosis treated with amphotericin B at the INI-Fiocruz between 1998 and 2018.

Variables	Category	HR (95%CI)	Adjusted HR (95% CI) ^a^
Bone involvement	No	2.31 (1.18–4.51)	2.81 (1.40–5.65)
Upper airway involvement	No	2.36 (1.17–4.75)	2.12 (1.05–4.27)
CNS involvement	No	2.74 (0.97–7.73)	2.70 (0.89–8.14)

^a^ Model adjusted by HIV, low treatment compliance and tuberculosis. HR = Hazard ratio; CI = Confidence interval.

## Data Availability

Not applicable.

## Supplementary Materials

The following supporting information can be downloaded at: https://www.mdpi.com/article/10.3390/jof8050469/s1, Figure S1: Morphological and molecular identification of *Sporothrix*.
