# Supplementary figures and images for: Severe Sporotrichosis Treated with Amphotericin B: A 20-Year Cohort Study in an Endemic Area of Zoonotic Transmission
Source: J Fungi (Basel). 2022 Apr 30;8(5):469. doi: 10.3390/jof8050469 (PMC9144044; doi:10.3390/jof8050469)

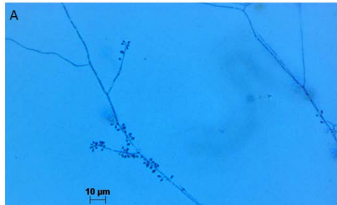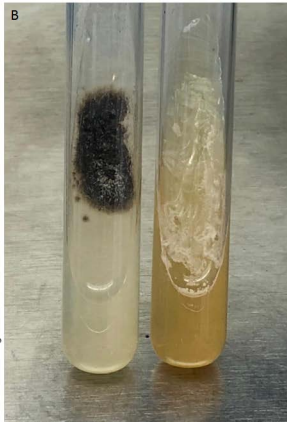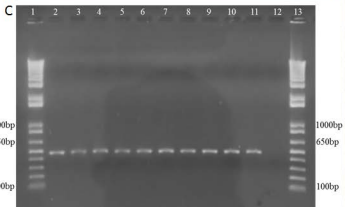

Supplement: Supplementary file 1 [file jof-08-00469-s001.zip › jof-1670683-supplementary Figure S1.pdf]
